# The Role of Diet, Additives, and Antibiotics in Metabolic Endotoxemia and Chronic Diseases

**DOI:** 10.3390/metabo14120704

**Published:** 2024-12-13

**Authors:** Ji-Eun Park, Ho-Young Park, Young-Soo Kim, Miri Park

**Affiliations:** 1 Food Functionality Research Division, Korea Food Research Institute, Jeonju 55365, Republic of Koreahypark@kfri.re.kr (H.-Y.P.); 2 Department of Food Science and Technology, Jeonbuk National University, Jeonju 54896, Republic of Korea; ykim@jbnu.ac.kr; 3 Department of Food Biotechnology, Korea National University of Science and Technology, Daejeon 34113, Republic of Korea

**Keywords:** diet, metabolic endotoxemia, chronic diseases, gut microbiota, intestinal permeability

## Abstract

**Background/Objectives**: Dietary patterns, including high-fat and high-carbohydrate diets (HFDs and HCDs), as well as non-dietary factors such as food additives and antibiotics, are strongly linked to metabolic endotoxemia, a critical driver of low-grade chronic inflammation. This review explores the mechanisms through which these factors impair intestinal permeability, disrupt gut microbial balance, and facilitate lipopolysaccharide (LPS) translocation into the bloodstream, contributing to metabolic disorders such as obesity, type 2 diabetes mellitus, and inflammatory bowel disease. **Methods**: The analysis integrates findings from recent studies on the effects of dietary components and gut microbiota interactions on intestinal barrier function and systemic inflammation. Focus is given to experimental designs assessing gut permeability using biochemical and histological methods, alongside microbiota profiling in both human and animal models. **Results**: HFDs and HCDs were shown to increase intestinal permeability and systemic LPS levels, inducing gut dysbiosis and compromising barrier integrity. The resulting endotoxemia promoted a state of chronic inflammation, disrupting metabolic regulation and contributing to the pathogenesis of various metabolic diseases. Food additives and antibiotics further exacerbated these effects by altering microbial composition and increasing gut permeability. **Conclusions**: Diet-induced alterations in gut microbiota and barrier dysfunction emerge as key mediators of metabolic endotoxemia and related disorders. Addressing dietary patterns and their impact on gut health is crucial for developing targeted interventions. Further research is warranted to standardize methodologies and elucidate mechanisms for translating these findings into clinical applications.

## 1. Introduction

The gastrointestinal tract represents the most extensive point of contact between an individual and their external surroundings within the body. Importantly, the gastrointestinal tract plays a crucial role in the selective absorption of essential nutrients from the intestines into the blood, and acts as a barrier to prevent the infiltration of damaging materials such as microorganisms, luminal antigens, and pro-inflammatory factors [1]. Western-style diets are high in fat and cholesterol, and are linked to increases in obesity and metabolic syndromes [2]. The composition and function of gut microbiota differ substantially across various sections of the gastrointestinal tract. The small intestine, where most nutrients are absorbed, has relatively low bacterial numbers due to its higher permeability and the rapid flow of luminal contents. In contrast, the large intestine houses over 99% of the microbiota, which play a central role in fermentation and the production of short-chain fatty acids (SCFAs) [3]. However, the large intestine also possesses a stronger gut barrier to prevent the translocation of harmful luminal molecules. This regional variation is essential to understanding how diet and microbiota interactions affect gut permeability and overall health [1,3].

Notably, these diets have been found to increase intestinal permeability and promote the release of lipopolysaccharide (LPS) into the bloodstream, leading to metabolic endotoxemia [4,5]. The studies reviewed used various methods to assess gut permeability, including the lactulose/mannitol ratio, which primarily reflects small intestine permeability, and FITC-dextran assays, often used for measuring large intestine permeability [6]. Each method has strengths and limitations, and results may differ depending on the region of the gut being studied [7]. Future research should carefully select methods that align with the specific intestinal region being investigated. However, variations in experimental designs, including differences in dietary compositions, animal models, and analytical techniques, pose challenges to the comparability of findings across studies. This highlights the urgent need for standardized methodologies to ensure reliability and consistency in assessing the impacts of dietary interventions on gut permeability and endotoxemia.

In this review, we have synthesized recent findings on the relationship between dietary interventions—including high-fat diets, high-carbohydrate diets, alcohol consumption, and food additives—and intestinal barrier dysfunction, with a particular focus on their role in modulating lipopolysaccharide (LPS) levels, promoting metabolic endotoxemia, and contributing to the pathogenesis of chronic metabolic disorders. By concentrating on these extensively studied dietary patterns, this review aims to elucidate their mechanistic impacts on gut microbiota composition, intestinal permeability, and systemic inflammation, while highlighting the necessity of future research to investigate additional dietary factors and their interactions with genetic, environmental, and lifestyle determinants.

## 2. Dietary Factors Influencing Endotoxemia

**Figure 1:**
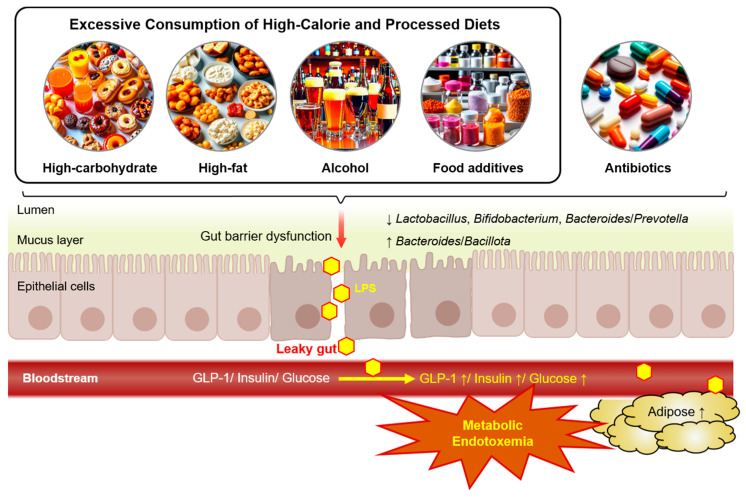
The potential mechanisms linking dietary factors to metabolic endotoxemia. Excessive and chronic consumption of high-carbohydrate diets, high-fat diets, alcohol, and food additives disrupt gut microbiota balance and impair gut barrier function, resulting in a leaky gut. Structural alterations to the intestinal epithelium caused by these dietary factors increase intestinal permeability, allowing lipopolysaccharides (LPSs) to translocate into the bloodstream. This LPS leakage triggers low-grade systemic inflammation, termed metabolic endotoxemia, which is associated with steatosis, insulin resistance, and adipocyte hypertrophy.

Contemporary Western diets are characterized by processed, stored, and transported foods, often with lower fiber contents than diets in developing nations and Western diets of the 1950s [8]. Recent research in animals and humans has highlighted a link between hypercaloric diets and metabolic endotoxemia [9,10]. In humans, intestinal fat absorption facilitates the uptake of endotoxins, including the potent pro-inflammatory agent lipopolysaccharide (LPS), which may contribute to or exacerbate inflammatory responses following a meal [4]. Notably, postprandial LPS levels increased significantly following the intake of high-fat meals compared to low-fat meals [4,11]. This review explores the potential mechanisms through which dietary factors, including high-carbohydrate diets (HCDs), high-fat diets (HFDs), alcohol, and food additives, contribute to metabolic endotoxemia (Figure 1). While these factors may have minimal effects when consumed in moderation or for short durations, prolonged or excessive intake has been implicated in the development of metabolic endotoxemia.

The field of microbiota research, especially in relation to metabolic endotoxemia, shows varying results on how diet affects microbial communities. These differences often stem from variations in study design, such as dietary composition, model types (human vs. animal), and microbial analysis methods [11,12]. For example, high-fat diet studies report both increases and decreases in certain bacterial populations, likely due to experimental or subject variability [8,13]. Further investigation is needed to understand the impact of factors like genetics, environment, and lifestyle. Standardized approaches in future research may help reconcile these differences.

### 2.1. High-Carbohydrate Diet (HCD)

The term “nutrient sugars” generally refers to neutral compounds composed of carbon, hydrogen, and oxygen, including sugars, oligosaccharides, and polysaccharides. The term “sugars” has been applied to simple carbohydrates, including monosaccharides and disaccharides. Oligosaccharides includes carbohydrates containing 3 to 10 sugars, and polysaccharides are complex carbohydrates such as starch, cellulose, and pectin. Although some carbohydrates are digestible in whole (sugars), others (indigestible fibers) represent completely indigestible nutrients [14]. Notably, contemporary diets are high in nutrient sugars (fructose and sucrose) and saturated fat. Increased calorie intake has been associated with various diet-related diseases, including cardiovascular diseases, metabolic syndrome, and nonalcoholic fatty liver disease (NAFLD). These findings are summarized in Table 1, which highlights the effects of high-carbohydrate diets on serum endotoxin levels, inflammatory cytokines, gut microbiota, and barrier function.

Fructose has emerged as a significant and prevalent component in contemporary Western diets [15]. A previous study showed that feeding C57BL/6J male mice 30% fructose for 8 weeks induced non-alcoholic fatty liver and liver inflammation, indicating that long-term intake of high fructose may disrupt lipid homeostasis [13]. Additionally, excessive intake of fructose has been linked to intestinal barrier dysfunction. Moreover, endotoxin levels were relatively higher following the intake of a high-fructose diet compared with that in the control group [16]. Furthermore, high-fructose diet intake significantly reduced the expression of tight junction (TJ) proteins that are responsible for maintaining mucosal barrier integrity, including zonula occludens (ZO)-1, occludin, mucin (Muc)-2, and Muc-4 [8,17]. Importantly, long-term intake of a high-fructose diet was associated with a decrease in the beneficial gut bacteria *Blautia*, *Ruminococcus*, *Bifidobacterium*, and *Lactobacillus*, and an increase in the *Bacillota/Bacteroides* ratio [18].

**Table 1:** A high-carbohydrate diet is an important factor in the development of many chronic diseases, serum endotoxin levels, inflammatory cytokines, gut barrier function, and changes in gut microbiota.

Diet Source	Sex	Animal	Duration (Week)	Disease	Microbiota	Inflammation Cytokines	Serum Endotoxin Level	Gut Barrier Function	Reference
50% Fructose	Male	C57BL/6 mice	12	Hepatic steatosis, NAFLD	Bacteroidetes ↓, Firmicutes, Proteobacteria, Actinobacteria ↑	-	LPS levels ↑	ZO-1, occludin ↓	[19]
30% Fructose	Female	C57BL/6j mice	12	Metabolic diseases	F/B ratio ↑	-	Endotoxin ↑	Gut permeability ↑, ZO-1 ↓	[8]
85% Glucose	Male	C57BL/6j mice	12	Obesity, hepatic steatosis	Bacteroidetes ↓, Proteobacteria ↑	TNF-α, IL-1β ↑	Endotoxin ↑	-	[20]
35% Fructose	Male	SD rats	8	Damage to the colonic barrier	Blautia, Ruminococcus, Lactobacillus ↓, Allobaculum ↑	IL-8, IL-6 ↑, IL-10 ↓	Endotoxin ↑	Claudin-4 ↓	[18]
2.31% Fructose	Male	C57BL/6 mice	8	NAFLD, hepatic fibrosis	Proteobacteria, Firmicutes ↑, Bacteroidetes ↓	IL-1β, IL-6 ↑	LPS levels ↑		[21]
60% Fructose	Male	SD rats	4	NAFLD	Bifidobacterium, Lactobacillus ↓	-	Endotoxin ↑	Occludin, claudin-1 ↓	[16]
20% Fructose solution (*w*/*v*) in drinking water	Male	Wistar rats	15	IBD	F/B ratio ↑, Actinobacteria ↓	TNF-α, IL-1β ↑	LPS levels ↑	TJP-2, occludin ↓	[22]
10.5 g/kg/day Fructose	Male	SD rats	20	Obesity, metabolic syndrome, diabetes mellitus	Lachnospira, Parasutterella, Marvinbryantia, Blantia ↑	IL-6, TNF-α, MIP-2 ↑, IL-10 ↓	-	ZO-1, occludin ↓	[23]
30% Fructose	Male	C57BL/6N mice	12	Neuroinflammation	Bacteroidetes ↓, Proteobacteria, Firmicutes ↑	IL-1β, TNF-α, IL-6 ↑	Endotoxin ↑	ZO-1, occludin, Muc2, Muc4 ↓	[24]
30% Fructose	Male	C57BL/6J mice	8	NAFLD	F/B ratio ↑, Bacteroidetes ↓	MCP-1, TNF-α ↑	Endotoxin ↑	Occludin, claudin-1 ↓	[13]
30% Fructose	Female	C57BL/6J mice	8	NAFLD	Bacteroidetes ↓	TNF-α, IL-1b ↑	LPS levels ↑	Gut permeability ↑, occludin, claudin-1 ↓	[17]
30% Fructose	Male	SD rats	8	Obesity, metabolic syndrome	Coprococcus, Ruminococcus, Clostridiaceae ↑	TNF-α, MPO ↑	LPS levels ↑	Occludin ↓	[25]
60% Fructose	Male	C57BL/6J mice	12	Dyslipidemia, insulin resistance, high blood pressure, hepatic steatosis NAFLD	Clostridium, Oscillospira Clostridiales ↑	TLR4 ↑	-	Muc2, occludin, claudin-2, claudin-5, ZO-1 ↓, Gut permeability ↑	[26]
20% Fructose	Male	Kunming mice	8	Obesity, NAFLD	Firmicutes, Epsilonbacteraeota, Bacteroidetes, Deferribacteres, Patescibacteria, Tenericutes ↓, Verrucomicrobia, Bacteroidetes ↑	-	-	Gut permeability ↑, ZO-1, occludin, Muc2 ↓	[27]
30% Fructose		C57BL/6 mice	11	Obesity	Proteobacteria, Bacteroidetes, Actinobacteria ↓	-	-	ZO-1, occludin, colon length, Muc2 ↓, Gut permeability ↑	[28]

SD, Sprague Dawley; NAFLD, nonalcoholic fatty liver disease; IBD, inflammatory bowel disease; F/B, *Firmicutes/Bacteroidetes*; TNF-α, tumor necrosis factor-α; IL-1β, *Interleukin*-*1β*; IL-8, *Interleukin-8*; IL-6, *Interleukin-6*; IL-10, *Interleukin-10*; MIP-2, Macrophage inflammatory protein-2; MCP-1, Monocyte Chemoattractant Protein-1; MPO, Myeloperoxidase; TLR4, toll-like receptor 4; LPS, lipopolysaccharide; ZO-1, zonula occludens-1; TJP-2, Tight junction protein 2; Muc-2, mucin 2; Muc-4, mucin 4. Arrows indicate observed trends: ↑ denotes an increase and ↓ denotes a decrease.

### 2.2. High-Fat Diet (HFD)

Lifestyle changes, particularly the adoption of Western diets, contribute to obesity. Research findings indicate that the intake of a HFD may cause endotoxemia [29]. A HFD significantly affects the metabolism of short-chain fatty acids (SCFA) and gut microbiota [30]. Notably, the gut microbiota plays a central role in human health and nutrition by facilitating nutrient absorption, preventing pathogen colonization, maintaining mucosal immunity, and regulating fat storage and metabolism [31]. These effects of HFDs on gut microbiota composition, inflammatory cytokines, serum endotoxin levels, and gut barrier function are comprehensively summarized in Table 2. A HFD alters the gut microbiota, leading to a significant decrease in specific types of Gram-negative and Gram-positive bacteria (*Lactobacillus*, *Bifidobacterium*, *Bacteroides–Prevotella*) and an increase in the proportion of *Bacteroides* and *Bacillota* [32]. *Bacteroidetes* and *Bacillota* are two key microbial communities that influence the body’s overall energy metabolism [33].

HFD intake induced structural alterations in the intestinal epithelium of experimental animals, resulting in an increase in LPS secretion into the bloodstream and plasma LPS levels, a condition known as metabolic endotoxemia. LPS-induced activation of toll-like receptor-4 (TLR4) triggers the production of various inflammatory cytokines, resulting in a state of low-grade systemic inflammation [9]. Research findings in mice indicate that HFD intake reduces the expression of epithelial TJ proteins such as claudins, occludin, and ZO-1 [34], contributing to intestinal hyperpermeability, which is linked to conditions such as diabetes, obesity, and inflammatory bowel disease (IBD) [35]. Additionally, HFD intake increased the levels of blood serum markers, such as total cholesterol, triglycerides (TG), low-density lipoprotein cholesterol, and fasting blood glucose, and decreased high-density lipoprotein cholesterol levels [34]. Overall, HFD intake triggers various metabolic disorders, such as cardiovascular disease, T2DM, and NAFLD, through mechanisms involving dysbiosis, compromised intestinal integrity, LPS absorption, and metabolic endotoxemia [36].

**Table 2:** A high-fat diet is an important factor in the development of many chronic diseases, serum endotoxin levels, inflammatory cytokines, gut barrier function, and changes in gut microbiota.

Diet Source	Sex	Animal	Duration (Week)	Disease	Microbiota	Inflammation Cytokines	Serum Endotoxin Level	Gut Barrier Function	Reference
37% Fat	Male	C57BL/6 mice	8	Obesity	Firmicutes, Thermotogae ↑, Proteobacteria ↓	TLR4, IL-6, IL-8, IL-10, TNF-α ↑	LPS levels ↑	_	[37]
60.08% Calories from fat	Male	C57BL/6J mice	8	Obesity	Bacteroidetes, S24-7, Porphyromonadaceae ↓, Lachnospiraceae, Ruminococcaceae, Rikenellaceae, Bacteroidaceae, F/B ratio ↑	TLR4, TNF-α, IL-6 ↑	LPS levels ↑	ZO-1, occludin ↓	[33]
60% Calorie high-fat diet	Male	C57BL/6J mice	14	Type 2 diabetes, Hyperglycemia	Clostridium septum ↑	TLR4, IL-6, MCP-1 ↑	Endotoxin ↑	_	[38]
60% kcal	Male	C57BL/6J mice	12	NAFLD	Firmicutes ↑	TNF-α ↑	LPS levels ↑	ZO-1, occludin, claudin-1 ↓	[39]
60 kcal% Fat	Male	C57BL/6J mice	8	Obesity	F/B ratio ↑	TNF-α, IL-1β, IL-6 ↑	LPS levels ↑	_	[40]
60 kcal% Fat	Male	C57BL/6J mice	9	Obesity	Allobaculum, F/B ratio ↑, Oscillospira ↓	TNF-α, IL-6 ↑, IL-10 ↓	LPS level ↑	_	[41]
45% Energy from fat	Male	C57BL/6J mice	12	Obesity	F/B ratio ↑	IL-6, TNF-α ↑	LPS levels ↑	Tjp1, occludin ↓	[42]
60% kcal Fat	Male	C57BL/6J mice	8	Obesity	F/B ratio ↑	IL-6, TNF-α ↑	LPS levels ↑	_	[43]
60% Energy for lard	Male	C57BL/6J mice	16	Obesity	Desulfovibrionaceae, Prevotellaceae, Verrucomicrobiaceae ↑	IL-1β, IL-10, TNF-α ↑	LPS, LBP levels ↑	_	[44]
45% kcal Fat	Male	BALB/c mice	10	Obesity	Firmicutes, Proteobacteria, Desulfovibrionales, Clostridia ↑, Bacteroidetes, Lactobacillale ↓	TNF-α ↑	LPS levels ↑	_	[45]
45% kcal Fat	Male	C57BL/6J mice	16	Metabolic disorders.	F/B ratio, Mucispirillum ↑, Turicibacter ↓	TNF-α ↑	LPS levels ↑	_	[46]
60% Calories from fat	Male	C57BL/6J mice	12	Obesity	Firmicutes ↑, Bacteroidetes ↓	TLR4, IL-1β, MCP-1 ↑	LPS levels ↑	_	[47]
60% Total calories from fat	Male	C57BL/6J mice	8	NAFLD		TLR4,TLR2, MCP-1, TNFα ↑	Endotoxin ↑	_	[48]
67% of Energy from fat	Male	Wistar albino rats	12	Metabolic disease	Bacteroidetes, Proteobacteria, Deinococcus-Thermus ↑	IL-6, TLR4, TNF-α ↑	LPS levels ↑	_	[49]
60% Fat calories	Male	C57BL/6 J mice	10	Intestinal infections, liver diseases, colon cancer, type II diabetes, obesity	Erysipelotrichaceae, Lactobacillaceae, Bifdobacterium, Lactobacillus ↓	TLR4, IL-6, IL-1β ↑	Endotoxin ↑	_	[50]
60% kcal Fat	Half male and half female	C57BL/6 mice	8	Obesity	Muribaculaceae, Bifidobacterium, Akkermansia ↓, Oscillospira, Lactococcus ↑	TNF-α, IL-6, IL-1β ↑	LBP levels ↑	_	[51]
45% Fat	Male	Wistar rats	6	Obesity, metabolic disorders.	F/B ratio ↑	IL-1β ↑	LBP levels ↑	Gut permeability ↑, Colon length, cecal weight, cecal and ileal crypt depth, goblet cells number ↓	[52]
45% High-fat diet	Male	C57BL/6 mice	24	Obesity	Bacteroidetes, Verrucomicrobiota ↓, F/B ratio, Dubosiella, Bifidobacterium, Faecalibaculum, Coriobacteriaceae ↑	TNF-α, MCP-1, IL-6, IL- 1β ↑	_	ZO- 1, claudin-2, occludin, MUC2 ↓	[34]
45% Energy from fat	Male	C57BL/6 mice	10	Hyperglycemia, hyperinsulinemia	F/B ratio ↑		LPS levels ↑	_	[53]
72% Fat	Male	C57BL/6J mice	4	Diabetes, obesity	*Lactobacillus* spp., *Bacteroides–Prevotella* spp. ↓		Endotoxin ↑	Gut permeability ↑, ZO-1, occludin ↓	[10]
60% kcal Fat	Male	C57BL/6 mice	6	Obesity		TNF-α ↑		Colon length, ZO-1, occludin, claudin, goblet cells ↓	[54]
60% of Calories from fat	Male	C57BL/6J mice	18	Obesity, type 2 diabetes	F/B ratio ↑	TNF-α ↑	_	ZO-1, claudin-1, occludin ↓	[55]

F/B, *Firmicutes/Bacteroidetes*; NAFLD, nonalcoholic fatty liver disease; TNF-α, tumor necrosis factor-α; IL-1β, *Interleukin*-*1β*; IL-8, *Interleukin-8*; IL-6, *Interleukin-6*; IL-10, *Interleukin-10*; MIP-2, Macrophage inflammatory protein-2; MCP-1, Monocyte Chemoattractant Protein-1; MPO, Myeloperoxidase; TLR2, toll-like receptor 2; TLR4, toll-like receptor 4; LPS, lipopolysaccharide; LBP, Lipopolysaccharide Binding Protein; ZO-1, zonula occludens-1; TJP-1, Tight junction protein 1; MCU-2, mucin 2; MUC-4, mucin 4. Arrows indicate observed trends: ↑ denotes an increase and ↓ denotes a decrease.

### 2.3. Alcohol

Alcohol consumption and metabolic syndrome are widely prevalent in populations worldwide and often coexist. Alcohol affects various bodily organs such as the liver, gut, and brain. Alcohol intake is particularly associated with various health issues, including chronic liver diseases, hepatocellular carcinoma, and liver-related outcomes such as decompensated cirrhosis or liver transplantation. The World Health Organization recommends that both men and women should not consume more than two standard drinks of pure ethanol per day [56]. Acute and chronic alcohol use have been shown to induce changes in intestinal barrier function in animals and humans [57]. Alcohol directly damages cells, and its major oxidative metabolite, acetaldehyde, alters the structure of intestinal epithelial TJ, resulting in increased intestinal permeability [6]. Disruption of intestinal barrier integrity can result in increased gastrointestinal permeability, potentially causing alterations in the gastrointestinal tract. Consequently, increased permeability can elevate systemic toxin levels in circulation, alter gut microbiota, and induce LPS production [9].

Additionally, increased release of pathogen-associated molecular patterns by microorganisms, such as lipoteichoic acid, activates Kupffer cells and promotes inflammatory signaling. Moreover, alcohol intake upregulated markers of liver damage, such as serum alanine aminotransferase, aspartate aminotransferase, and neutrophilic TG, in mice [58]. Positive correlations have been established between serum LPS levels and alcohol consumption levels [59]. Therefore, it is essential to regulate alcohol intake to prevent LPS production.

### 2.4. Additives in Processed Foods

Recently, there has been growing awareness among consumers and health professionals about the potential health risks associated with consuming processed foods containing emulsifiers and preservatives. Emulsifiers and preservatives in processed foods disrupt gut microbiota and intestinal barrier function, contributing to metabolic endotoxemia. Emulsifiers are chemicals that help blend ingredients in processed foods, while preservatives are added to extend the shelf life of products. Research suggests that these additives can negatively affect gut health and contribute to metabolic dysfunction. For example, Chassaing et al. showed that common dietary emulsifiers (such as carboxymethylcellulose and polysorbate-80) altered gut microbiota composition, increased intestinal permeability, and promoted low-grade inflammation, leading to metabolic syndrome and colitis in susceptible mice [60].

Similarly, preservatives in processed foods have been implicated in gut dysbiosis and metabolic disturbances [61]. Notably, the intake of diets rich in preserved foods containing additives, such as sodium benzoate and sodium nitrite, altered gut microbiota diversity and composition following a preserved foods diet [62,63]. Overall, these findings highlight the potential role of emulsifiers and preservatives in disrupting gut microbiota and intestinal barrier function, leading to metabolic endotoxemia and associated metabolic disorders.

### 2.5. Antibiotics

Antibiotics are not considered dietary, although they can contribute to metabolic endotoxemia by increasing the quantity of bacterial endotoxin in the bloodstream. Antibiotics can cause dysbiosis, resulting in increased intestinal permeability and translocation of bacterial endotoxins into the bloodstream. Cox et al. [64] found that antibiotic-induced changes in gut microbiota composition increased intestinal permeability and induced metabolic endotoxemia, leading to metabolic dysfunction and obesity in mice. Additionally, antibiotic exposure induced changes in gut microbiota composition, increased intestinal permeability, and caused metabolic endotoxemia, ultimately leading to obesity and metabolic syndrome in mice [65]. A study on obese (*ob*/*ob*) mice showed that antibiotic treatment reduced gut microbial population, leading to a significant decrease in beneficial bacteria such as *Lactobacillus*, *Bifidobacterium*, and *Bacteroides*–*Prevotella* species [10]. Although antibiotic treatment ameliorated metabolic endotoxemia in the mice, their LPS levels remained high. Collectively, these findings suggest that antibiotics can induce metabolic endotoxemia by altering the gut microbiota composition. Additionally, antibiotic treatment downregulated the expression of inflammatory markers (PAI-1 and F4/80) in adipose tissue, reduced oxidative stress (lipid peroxides), and improved metabolic parameters (glucose intolerance and insulin resistance) in *ob*/*ob* mice. Overall, these findings indicate that antibiotic-induced dysbiosis may disrupt intestinal barrier function, allowing bacterial endotoxins to move into the bloodstream and induce metabolic endotoxemia and inflammation, oxidative stress, and metabolic dysregulation.

Furthermore, there is a growing body of evidence linking antibiotic use to alterations in gut microbiota and metabolic health in humans. Longitudinal studies such as the *Framingham Heart Study* and *the European Prospective Investigation into Cancer and Nutrition* (EPIC) have reported associations between antibiotic use and increased risk of obesity and metabolic syndrome. Although antibiotics play a crucial role in treating bacterial infections, indiscriminate use can have unintended consequences on gut microbiota composition and metabolic health, potentially contributing to metabolic endotoxemia and associated metabolic disorders [66].

## 3. Impact of Diet-Induced Endotoxemia on Health Outcomes

The human intestinal lumen provides a vast surface for bacterial colonization, which can potentially lead to toxin production. The intestinal epithelium serves as a vital barrier, preventing the absorption of LPS, a notable toxin [67]. A healthy intestinal barrier facilitates the passage of water, nutrients, and beneficial bioactive compounds, and obstructs harmful substances such as microbial and dietary antigens [68]. However, LPS can enter the bloodstream when the intestinal mucosa is compromised, resulting in endotoxemia. Notably, endotoxemia manifests symptoms that affect organ and cellular structure and function, altering metabolic processes, increasing body temperature, changing hemodynamics, and potentially leading to septic shock [68,69]. LPS is a substance found in the cell membranes of Gram-negative bacteria, and comprises three components: a variable O-antigen composed of repeating oligosaccharide units, a core oligosaccharide, and lipid A [68].

**Figure 2:**
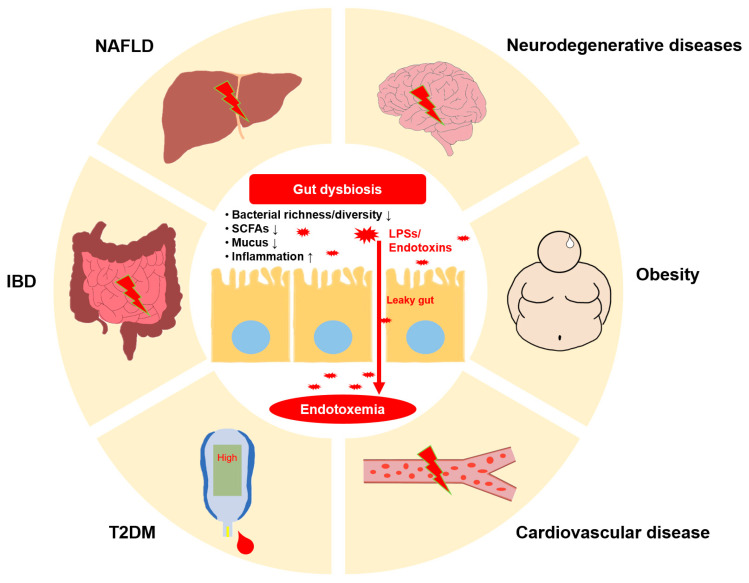
The impact of dietary factors on gut health and systemic diseases: Excessive consumption of high-fat or high-carbohydrate diets, along with alcohol and food additives, disrupts gut microbiota composition, leading to dysbiosis and increased intestinal permeability. This allows lipopolysaccharides (LPS) and other pro-inflammatory molecules to translocate into the bloodstream, inducing chronic low-grade inflammation. These processes contribute to the development of metabolic diseases such as obesity and type 2 diabetes mellitus (T2DM), liver dysfunction such as nonalcoholic fatty liver disease (NAFLD), and inflammatory conditions like inflammatory bowel disease (IBD). Additionally, systemic inflammation is linked to the onset of neurodegenerative diseases and cardiovascular diseases, highlighting the widespread health consequences of dietary imbalances.

LPS triggers the release of inflammatory mediators, such as tumor necrosis factor, interleukin-6, and platelet-activating factor, once absorbed into circulation [29,69,70]. As described in Figure 2, the importance of dietary regulation cannot be overemphasized, as diet-induced conditions play a significant role in the development of various chronic diseases characterized by low-grade inflammation, such as obesity, insulin resistance (IR), type 2 diabetes mellitus (T2DM), cardiovascular disease, and dyslipidemia, in both animals and humans [9,71]. This series of events triggers a widespread inflammatory reaction that is associated with serious conditions such as acute respiratory syndrome, cancer, extensive burns, and acute peritonitis.

It is important to acknowledge the challenges involved in accurately measuring serum lipopolysaccharide (LPS) levels, particularly the potential for false positives due to β-glucan interference, which has been a significant issue in the field. β-glucans, commonly present in fungal cell walls, can trigger false-positive results in LPS assays, complicating the interpretation of endotoxemia data [69,70]. While the studies discussed provide valuable insights, many may not account for this issue. Future research should aim to use validated, endotoxin-specific assays to improve accuracy in LPS quantification.

### 3.1. Inflammatory Bowel Disease (IBD)

IBD encompasses a group of disorders characterized by severe inflammation of the gastrointestinal tract, presenting symptoms such as abdominal pain, diarrhea, and weight loss. IBD affects approximately 1 in 250 individuals in the European population, as well as a significant number of individuals from diverse ethnic backgrounds. The etiology of IBD remains unknown, leading to poor prognoses and lifelong morbidity in affected patients. IBD is primarily classified into two categories: ulcerative colitis (UC) and Crohn’s disease (CD) [72]. CD is a severe inflammatory disorder of the immune system that can affect any part of the gastrointestinal system, with its causative factors still unidentified. UC is a chronic condition that primarily affects the mucosa of the large intestine, and is characterized by patterns of inflammation and ulceration that alternate between relapse and remission without apparent triggers [73]. Research suggests that factors, such as high-fat diets, may contribute to the increased prevalence of IBD [74].

The human gut microbiota, comprising over 100 trillion microbial cells, has been associated with gastrointestinal diseases such as IBD [73]. Genetic defects in patients with IBD can disrupt the composition of the microbiota, potentially compromising the beneficial effects of certain microbes on host immunity [75]. In UC, there is a decrease in *Lactobacillus* and *Bifidobacterium* abundance, and an increase in *Bacteroides vulgatus* and *Fusobacterium* abundance. Similarly, CD is linked to decreased levels of beneficial microbial species, such as *Lactobacillus* and *Bifidobacterium*, as well as *Faecalibacterium prausnitzii*, which produces anti-inflammatory metabolites [76]. Notably, there is a consistent decrease in *Bacillota* in IBD patients, along with reduced levels of SCFA-producing microorganisms such as *Clostridium* spp. SCFA deficiency is positively correlated with disease progression, indicating the potentially beneficial role of SCFAs in maintaining intestinal homeostasis through various mechanisms [74,75]. The etiology of IBD is believed to involve intricate interactions among the host, gut microbes, and diet [74]. In addition to genetic factors, the balance of intestinal flora is thought to play a crucial role in the pathogenesis of these diseases [73,74]. Although existing studies have elucidated the complex interplay between diet, gut microbiota, and host in the context of IBD, further research is necessary to elucidate the precise underlying causes.

### 3.2. Type 2 Diabetes Mellitus (T2DM)

T2DM is a complex chronic condition characterized by varying levels of IR and impaired insulin secretion. Currently, the global prevalence of diabetes exceeds 415 million individuals, and it is projected to increase to 642 million by 2040. T2DM, a prevalent metabolic disorder, is linked to numerous long-term complications such as nephropathy, angiopathy, retinopathy, and peripheral neuropathy [50]. Chronic low-grade systemic inflammation is recognized as a contributing factor to the development of T2DM, with risk modification influenced by factors including age, gender, ethnicity, genetics, and dietary habits [77]. A study conducted on rodents showed that the intake of high-sugar and HFD regimen for 48 weeks induced T2DM, resulting in obesity, dyslipidemia, hyperglycemia, glucose intolerance, and IR. Notably, these animals exhibited elevated levels of circulating LPS, tumor necrosis factor (TNF)-α, interleukin (IL)-6, and ALP in their intestinal tissue homogenates after week 12, indicating the onset of intestinal endotoxemia and chronic inflammation that persisted throughout the 48 weeks [67]. Clinical and experimental evidence suggest a potential role of the intestines in the progression of T2DM, with diabetic patients and certain T2DM animal models showing significantly increased intestinal permeability [10,78]. Recent research suggests that prolonged intake of a HFD may contribute to the development of T2DM by altering the composition of intestinal microbiota, leading to an increase in Gram-negative bacteria associated with compromised intestinal barrier function. Overall, this alteration may facilitate the chronic absorption of LPS and other toxins, ultimately triggering metabolic endotoxemia [79].

### 3.3. Obesity

Obesity is a condition characterized by excess accumulation of fat beyond normal levels, resulting from an intake of fat that surpasses the body’s capacity for neutral fat storage [35,43,80]. Notably, the increasing prevalence of obesity is recognized as a significant social and public health concern globally [80,81]. The World Health Organization defines overweight as having a Body Mass Index (BMI) exceeding 25 kg/m^2^, and obesity as a BMI exceeding 30 kg/m^2^ [82]. Obesity exerts significant effects on cardiovascular diseases, IR, hypertension, atherosclerosis, and the development of T2DM [31,82]. Obesity is characterized by adipocyte proliferation and hypertrophy and increased secretion of pro-inflammatory cytokines (TNF-α and IL-6) by adipose tissue macrophages [83]. Although the specific pathogenesis of obesity remains poorly understood, emerging evidence indicates that the gut microbiota plays a crucial role in regulating energy metabolism and the digestion of essential nutrients such as carbohydrates, proteins, and vitamins [34,35,84]. Specifically, a reduction in beneficial bacterial species, such as *Bifidobacterium*, *Lactobacillus*, and *Akkermansia muciniphila*, has been linked to the development of obesity in both humans and mice. Moreover, alteration in *Bacillota/Bacteroidetes* ratio is associated with elevated glucose levels and body weight [53,57]. Furthermore, imbalance in intestinal microbiota is correlated with an increase in pathogenic bacteria, resulting in endotoxemia, damage to intestinal cells, and disruption of intestinal permeability [35]. LPS-induced alteration in intestinal barrier function and integrity decreases the levels of the key TJ proteins ZO-1, occludin, and claudin1, resulting in increased intestinal permeability and possibly obesity [25,33].

### 3.4. Cardiovascular Disease

Endotoxins can cause systemic inflammation, which promotes endothelial dysfunction, atherosclerosis development, and thrombosis, raising the risk of cardiovascular events such as myocardial infarction and stroke [85]. Particularly, chronic low-grade inflammation induced by endotoxemia plays a pivotal role in the initiation and progression of atherosclerosis, a condition characterized by the buildup of plaque in arterial walls. Inflammatory mechanisms stimulate immune cell recruitment and the creation of fatty streaks, which eventually develop into atherosclerotic plaques. These plaques can narrow arteries, restrict blood flow, and increase the risk of myocardial infarction and stroke. Notably, individuals with high endotoxin levels showed considerably elevated risk (up to three times) of developing atherosclerosis and cardiovascular disease over 5–10 years of follow-up, even after accounting for conventional risk factors [86]. Inflammation-induced endothelial dysfunction, oxidative stress, and changes in renal function all contribute to the development of hypertension. Additionally, endotoxemia may stimulate the sympathetic nervous system and the renin–angiotensin–aldosterone system, further exacerbating hypertension. Dietary imbalance-induced endotoxemia can disrupt lipid metabolism, leading to dyslipidemia characterized by elevated levels of triglycerides and LDL cholesterol and reduced levels of HDL cholesterol. Dyslipidemia causes atherosclerosis by allowing cholesterol-rich lipoproteins to accumulate in artery walls, contributing to plaque formation and development [87]. Inflammatory cytokines and oxidative stress can directly damage cardiac tissue and impair cardiac function, resulting in heart failure [88]. Conclusively, persistent inflammation induced by endotoxemia can damage blood vessels and increase plaque formation, leading to endothelial dysfunction and the development of atherosclerosis, increasing the risk of cardiovascular disease such as atherosclerosis and stroke.

### 3.5. Nonalcoholic Fatty Liver Disease (NAFLD)

NAFLD is a condition in which fat accumulates in the liver (hepatic steatosis) in individuals with high alcohol intake. Endotoxemia leads to NAFLD progression by causing chronic low-grade inflammation, lipid buildup, and fibrogenesis in the liver. The portal vein connects the gut to the liver, establishing the gut–liver axis [89]. Blood endotoxin levels are higher in individuals with simple steatosis than in healthy individuals, and even higher in those with non-alcoholic steatohepatitis [90]. High endotoxin levels are associated with severe hepatic steatosis, inflammation, ballooning, and fibrosis grades on liver histology [91]. Endotoxins stimulate the TLR4 signaling pathway in hepatocytes and Kupffer cells, resulting in inflammatory cytokine production and hepatocyte damage [90]. Increased hepatic expression of CD14 (endotoxin co-receptor) might contribute to endotoxin hyper-responsiveness in NAFLD [92]. Endotoxemia causes inflammatory responses by activating TLR4, resulting in the production of pro-inflammatory cytokines such as TNF-α and IL-6 [12]. Oxidative stress in NAFLD is exacerbated by reactive oxygen species (ROS) produced during fatty acid metabolism in the liver as well as inflammation. Oxidative stress causes lipid peroxidation, mitochondrial dysfunction, and hepatocyte injury, which exacerbates liver damage and inflammation [93]. Several clinical trials have shown a link between endotoxemia and NAFLD severity. Increased serum LPS levels and endotoxin exposure markers have been linked to the development and progression of NAFLD, as well as insulin resistance and systemic inflammation [94]. Overall, endotoxemia plays a significant role in the pathogenesis and progression of NAFLD through chronic inflammation, steatosis, and fibrosis in the liver.

### 3.6. Neurodegenerative Diseases

Endotoxemia, or the presence of endotoxins in the bloodstream, has been linked to various neurological conditions, including neurodegenerative diseases such as Alzheimer’s. Importantly, blood and brain endotoxin levels are higher in patients with Alzheimer’s disease than in healthy individuals. Systemic infections, such as periodontal disease, can worsen Alzheimer’s disease by increasing endotoxin levels. Additionally, systemic inflammation can promote neuroinflammation by activating microglia, the central nervous system’s resident immune cells, and increasing the synthesis of inflammatory mediators within the brain [95]. Endotoxemia-induced inflammation can jeopardize the blood–brain barrier (BBB), a selective barrier that controls the flow of chemicals between the bloodstream and the brain. The disruption of the BBB permits circulating inflammatory cytokines and LPS to reach the brain, increasing neuroinflammation and neuronal injury [96]. Neuroinflammation and BBB disruption both contribute to neuronal dysfunction and neurodegeneration in several neurological conditions. Chronic exposure to inflammatory mediators and oxidative stress can impair synaptic function, induce neuronal death, and contribute to the development of diseases such as Alzheimer’s disease, Parkinson’s disease, and multiple sclerosis [97]. Animal studies have shown that administering LPS or inducing endotoxemia can cause neuroinflammation, cognitive impairments, and behavioral abnormalities similar to neurological diseases. Overall, these investigations offer mechanistic insights into the involvement of endotoxemia in the etiology of neuroinflammatory and neurodegenerative diseases [98].

Although it has been suggested that endotoxemia-induced neuroinflammation may contribute to the pathophysiology of Alzheimer’s disease and possibly other neurodegenerative illnesses, studies are necessary to establish causality and develop treatment strategies targeting endotoxemia. Clinical studies have found higher serum levels of LPS and inflammatory markers in individuals with Alzheimer’s disease, Parkinson’s disease, and multiple sclerosis, indicating a possible relationship between endotoxemia, systemic inflammation, and neurological disorders in humans [99]. Conclusively, endotoxemia contributes to neuroinflammation, neuronal dysfunction, and cognitive impairment in various neurological disorders.

## 4. Conclusions and Future Perspectives

In this literature review, we outline the findings of recent research on the effects of HFDs and HCDs, chronic diseases associated with alcohol consumption, serum endotoxin levels, and key factors influencing the production of inflammatory cytokines. Additionally, we explored the effects of HFDs and HCDs on intestinal barrier function and changes in the intestinal microbiota. Research findings indicate that the intake of HFDs and HCDs can induce endotoxemia, resulting in structural changes in the intestinal epithelium and intestinal mucosal damage. Additionally, intestinal microbiota-derived LPS plays a role in the pathogenesis of obesity and related disorders in animal models by elevating systemic endotoxin levels and disrupting intestinal microbiota. Notably, disruption in intestinal microbiota leads to a low-grade inflammatory state with systemic implications due to compromised intestinal barrier function. The intricate relationship between health and nutrition primarily unfolds in the intestine, underscoring the critical importance of intestinal barrier function. Additionally, while animal studies provide valuable insights into the mechanisms of diet-induced metabolic endotoxemia, human studies are crucial to confirm these findings and translate them into practical applications. Future research should focus on addressing this gap to enhance our understanding of the relevance of these mechanisms in human health.

## Data Availability

The original contributions presented in this study are included in the article. Further inquiries can be directed to the corresponding author.
